# Fitz‐Hugh‐Curtis syndrome without salpingitis: Should contrast‐enhanced computed tomography be a routine diagnostic procedure?

**DOI:** 10.1002/ccr3.5211

**Published:** 2021-12-13

**Authors:** Taku Harada, Taro Shimizu

**Affiliations:** ^1^ Division of General Medicine Showa University Koto Toyosu Hospital Tokyo Japan; ^2^ Division of Diagnostic and Generalist Medicine Dokkyo Medical University Hospital Tochigi Japan

**Keywords:** clinical diagnosis, Fitz‐Hugh‐Curtis syndrome, pelvic inflammatory disease, risk from radiation exposure

## Abstract

Fitz‐Hugh‐Curtis syndrome should be suspected in sexually active young women presenting with acute right upper quadrant pain even in the absence of lower abdominal pain. The risk of radiation exposure associated with computed tomography should also be considered while making a diagnosis of abdominal pain in young patients.

## Graphical abstract

Fitz‐Hugh‐Curtis syndrome should be suspected in sexually active young women who present with acute right upper quadrant pain without lower abdominal pain, and the risk of radiation exposure should be considered when performing computed tomography.

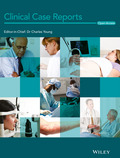

## 1 INTRODUCTION

Fitz‐Hugh‐Curtis syndrome (FHCS), also known as perihepatitis, is a manifestation of chronic pelvic inflammatory disease (PID), with an incidence rate of 4%–27%.^1^ Clinically, FHCS generally presents as lower abdominal pain, with or without accompanying upper quadrant abdominal pain, and suprapubic tenderness, which is present in more than 95% of cases of PID.^2^ Although salpingitis is generally associated with FHCS, cases of FHCS without salpingitis have previously been reported, although such cases are infrequent.^3^, ^4^ The diagnosis of FHCS in the absence of salpingitis is challenging,^4^, ^5^ and the resulting delay in diagnosis can possibly lead to intra‐abdominal adhesion, subdiaphragmatic abscess, small bowel obstruction, and subsequent unnecessary open surgery.^1^, ^3^ Perihepatic enhancement by contrast‐enhanced computed tomography (CT) can be useful for the diagnosis of FHCS.^6^ However, there is currently no research comparing the diagnostic benefits of CT and its associated risks of radiation exposure. Herein, we present a case of FHCS without salpingitis in a 15‐year‐old girl who presented with only upper abdominal pain and pleuritic pain. The diagnosis of FHCS was based on the history and clinical examination, without the need for CT.

## 2 CASE DESCRIPTION

A 15‐year‐old girl, with no significant past medical history, presented to the emergency department with acute upper right quadrant pain of two days duration. The pain was aggravated by coughing, deep inspiration, right lateral position, and general body movements and radiated to the right shoulder. She was sexually active, with only one specific partner in her history. She did not use any protection against sexually transmitted infection (STI) or for pregnancy prevention. At the time of assessment, the patient was 23 days from her last menstrual period and was unsure of her pregnancy status. The patient denied the presence of vomiting, diarrhea, bloody stool, fever, chest pain, vaginal discharge, and lower back pain. She reported a transient episode of atypical genital bleeding a few days prior, which was not present at the time of the visit.

On physical examination, she appeared unwell with a body temperature of 37.5°C, blood pressure of 113/71 mm Hg, pulse of 109 bpm, respiratory rate of 22 breaths/min, and O_2_ saturation of 96% at normal room conditions. Her neck was supple. On auscultation, heart sounds were unremarkable, and lungs were clear bilaterally. There was knocking tenderness above the liver and right costovertebral angle. Peritoneal signs (coughing test and percussion tenderness) were positive but limited to the right upper quadrant. Rebound tenderness was not performed because the peritoneal signs were apparent with the aforementioned less‐invasive techniques. No tenderness was elicited in the lower abdominal (including McBurney's and Lanz's points) and pelvic areas. Blood analysis did not reveal any significant abnormality, except for an elevated white blood cell count (16.6×10^9^/L) and C‐reactive protein level (71.7 mg/L). the liver function tests were normal. Urine analysis for human chorionic gonadotropin and Gram staining for bacteria revealed negative results. No abnormality was noted on chest radiographs and abdominal ultrasound. Considering the above information, we suspected FHCS without salpingitis. Laboratory tests for hepatitis virus, human immunodeficiency virus, and syphilis revealed negative results. Blood culture, urine culture, and polymerase chain reaction (PCR) analysis for uterocervical gonococcus and chlamydia were performed, with positive results for chlamydia and negative for gonococcus. Azithromycin and ceftriaxone treatment was initiated for suspected FHCS and/or urinary tract infection. The symptoms were subsided on the day following treatment initiation. Her sexual partner was diagnosed and treated for chlamydia infection.

## 3 DISCUSSION

Our patient was diagnosed with FHCS without salpingitis. Generally, suprapubic tenderness is present in more than 95% cases of PID,^2^ which was not reported by our patient. FHCS can occur without salpingitis and, although such cases are clinically challenging to diagnose, early diagnosis is crucial to avoid for related complications.

It is not clear how often salpingitis accompanies FHCS. In the past, FHCS was defined as salpingitis with perihepatitis,^7^ and FHCS without lower abdominal pain was considered a rare occurrence.^1^ A study by You et al.^8^ on the clinical manifestation of FHCS in the emergency department reported that 55% of patients presented with right upper quadrant pain only, and only 33% of patients complained of lower quadrant pain along with right upper quadrant pain.^8^ However, 91% of patients had pelvic fat infiltration on CT imaging. It is possible that lower abdominal symptoms and findings may have been underestimated in this retrospective study. In any case, it is important to suspect FHCS among sexually active young women presenting with an acute onset right upper quadrant pain or pleuritic pain, regardless of the presence or absence of lower abdominal symptoms.^5^

There are reports of atypical FHCS presenting with right shoulder pain or left upper quadrant pain.^9^, ^10^ In high‐risk patients, to consider FHCS as the differential diagnosis, a thorough medical history is essential, and it is advisable to include a detailed history of sexual activity, including the Five P's: partners, practices, pregnancy prevention, protection against sexually transmitted diseases (STDs), and past history of STDs.^11^ Privacy concerns need to be considered when taking a detailed history of sexual activity and, therefore, providing an explanation of the importance of this history for diagnosis needs to be carefully explained. In addition, if an STI is diagnosed, screening for other STIs is needed, including screening and treatment of the partner. The recurrence rate of FHCS when the partner is untreated has been reported at 14%–39%.^12^

Plain abdominal radiographs contribute only a small amount of radiation exposure but radiographs are of limited value for diagnosis of diseases involving the right upper quadrant. Radiographs are useful in cases where the cause of symptoms is thought to be in a location other than the right upper quadrant, such as pneumonia, pleural effusion, gastrointestinal perforation, or intestinal obstruction.^3^ Abdominal ultrasound is a useful technique for exclusion of conditions, which present with upper quadrant pain resembling FHCS, such as cholecystitis. However, it is not a standard test for the diagnosis of FHCS and its diagnostic precision is unknown.^1^ If acute right upper quadrant pain is suspected to be biliary tract disease but the ultrasound image is negative or equivocal, magnetic resonance cholangiopancreatography (MRCP) or contrast‐enhanced CT is recommended.^6^ Although FHCS is not a biliary tract disease, perihepatic enhancement by contrast‐enhanced CT is reported to be useful for its diagnosis. However, the reported sensitivity of contrast‐enhanced CT is only 55.9%. It is necessary to consider the risk of radiation associated with CT when making decisions regarding diagnostic imaging for young patients.^13^, ^14^ There are no documented standard diagnostic criteria for FHCS and the diagnosis of perihepatitis itself is made by invasive diagnostic techniques of laparoscopy or laparotomy. Surgical intervention for FHCS is warranted only if symptoms do not resolve with therapy.^1^

The risk of radiation‐induced malignancy from abdominal CT for a 15‐year‐old girl is approximately 0.15%, with >0.1% of radiation‐associated malignancies after the age of 25 years.^13^ Smith‐Bindman et al.^14^ calculated that 250 multiphase abdominal and pelvic CT scans in a 20‐year‐old woman would result in development of one cancerous tumor. Therefore, it is necessary to consider risks and benefits associated with radiation exposure before performing CT imaging for adolescents. MRCP, which has no risks of radiation, is recommended for evaluation of biliary tract diseases.^6^

The question that needs to be asked is as follows: Should a contrast‐enhanced CT be a routine diagnostic procedure for suspected FHCS? Although contrast‐enhanced CT is a helpful diagnostic aid, it carries the risk of radiation exposure and is not sensitive enough to accurately diagnose FHCS. Therefore, we believe that, in a young patient presenting with only right upper quadrant pain in the presence of a sexually transmitted disease, FHCS without salpingitis should be considered as a possible diagnosis and empirical therapy must be initiated without performing a CT. Chlamydia is more frequent than gonorrhea as a cause of FHCS and uterocervical PCR is the best test for both microorganisms.^1^

Thus, we conclude that CT is not necessary for diagnosing FHCS. Careful history taking, detailed physical examination, and uterocervical bacterial testing are sufficient to make this diagnosis. Physicians should always consider FHCS as a possible diagnosis in sexually active young women complaining of acute right upper quadrant pain even in the absence of lower abdominal symptoms.

## CONFLICT OF INTEREST

The authors declare no competing interests.

## AUTHOR CONTRIBUTIONS

Taku Harada, Taro Shimizu: Conception and design. Taku Harada: Acquisition of data. Taku Harada: Patient management and interpretation of data. Taku Harada, Taro Shimizu: Manuscript writing.

## CONSENT

The written informed consent was obtained from the patient and her family to publish this report in accordance with the journal's patient consent policy.

## Data Availability

Data sharing not applicable – no new data generated.

## References

[1] Peter NH , Clark LR , Jaeger JR . Fitz‐Hugh‐Curtis syndrome: a diagnosis to consider in women with right upper quadrant pain. Clevel Clin J Med. 2004;71(3):233‐239. 10.3949/ccjm.71.3.233 15055246

[2] Brunham RC , Gottlieb SL , Paavonen J . Pelvic inflammatory disease. N Engl J Med. 2015;372(21):2039‐2048. 25992748 10.1056/NEJMra1411426

[3] Counselman FL . An unusual presentation of Fitz‐Hugh‐Curtis syndrome. J Emerg Med. 1994;12(2):167‐170. 8207151 10.1016/0736-4679(94)90694-7

[4] Katzman DK , Friedman IM , McDonald CA , Litt IF . Chlamydia trachomatis Fitz‐Hugh‐Curtis syndrome without salpingitis in female adolescents. Am J Dis Child. 1988;142(9):996‐998. 3414633 10.1001/archpedi.1988.02150090094033

[5] Mitaka H , Kitazono H , Deshpande GA , Hiraoka E . Fitz‐Hugh‐Curtis syndrome lacking typical characteristics of pelvic inflammatory disease. BMJ Case Rep. 2016;2016:bcr2016215711. 10.1136/bcr-2016-215711 PMC4932418 27335367

[6] Kim JY , Kim Y , Jeong WK , Song SY , Cho OK . Perihepatitis with pelvic inflammatory disease (PID) on MDCT: characteristic findings and relevance to PID. Abdom Imaging. 2009;34(6):737‐742. 19002524 10.1007/s00261-008-9472-9

[7] Ris HW . Perihepatitis (Fitz‐Hugh–Curtis syndrome). A review and case presentation. J Adolesc Health Care. 1984;5(4):272‐276. 6436214 10.1016/s0197-0070(84)80131-x

[8] You JS , Kim MJ , Chung HS , et al. Clinical features of Fitz‐Hugh‐Curtis syndrome in the emergency department. Yonsei Med J. 2012;53(4):753‐758. 22665342 10.3349/ymj.2012.53.4.753 PMC3381477

[9] Khine H , Wren SB , Rotenberg O , Goldman DL . Fitz‐Hugh‐Curtis syndrome in adolescent females: a diagnostic dilemma. Pediatr Emerg Care. 2019;35(7):e121‐123. 29794956 10.1097/PEC.0000000000001525

[10] Tada N , Ishizuka K , Yokokawa D , Ikusaka M . Fitz‐Hugh‐Curtis syndrome with right shoulder pain. Postgrad Med J. 2021:postgradmedj‐2021‐140985. 10.1136/postgradmedj-2021-140985 34413178

[11] Workowsk KA , Bolan GA . Centers for disease control and prevention: Sexually transmitted diseases treatment guidelines, 2015. Morb Mortal Wkly Rep. 2015;64(RR3):1‐137.

[12] Gaydos CA , Wright C , Wood BJ , Waterfield G , Hobson S , Quinn TC . Chlamydia trachomatis reinfection rates among female adolescents seeking rescreening in school‐based health centers. Sex Transm Dis. 2008;35(3):233‐237. 18490866 10.1097/OLQ.0b013e31815c11fe PMC2664683

[13] Brenner D , Eliston C , Hall E , Berdon W . Estimated risks of radiation‐induced fatal cancer from pediatric CT. Am J Roentgenol. 2001;176(2):289‐296. 11159059 10.2214/ajr.176.2.1760289

[14] Smith‐Bindman R , Lipson J , Marcus R , et al. Radiation dose associated with common computed tomography examinations and the associated lifetime attributable risk of cancer. Arch Intern Med. 2009;169(22):2078‐2208. 20008690 10.1001/archinternmed.2009.427 PMC4635397

